# Valedictory

**Published:** 1855-12

**Authors:** 


### VALEDICTORY.

With the present number our connection with the *Western Journal of Medicine and Surgery* ceases, and the work, under another name, passes into the hands of other conductors. From the subjoined circular it will be seen that it is to be edited, in future, by Prof. Gross and Dr. T. G. Richardson.

We have been for a good while desirous of retiring from the post of editor, which we felt that we were occupying too long, but never saw our way quite clear till now. It is nearly twenty-five years since Kentucky became our home, and of that time we have been connected twenty-three years with its medical press. In an address delivered by Prof. Drake to the Medical Library Association of Cincinnati, four years ago, our lamented colleague spoke of us as then "the senior Medical Editor of the Interior Valley." Whatever may be the advantages derived from long experience in this line of labor, and we are not disposed to deny or under-value them, they are hardly equivalent to the zeal and ardor with which we naturally enter new enterprises, but which infallibly abate in the progress of years.

When at the request of our colleagues we took charge of the *Transylvania Journal of Medicine and the Associate Sciences*, towards the close of the year 1831, there was but one other medical journal published in the Valley of the Mississippi—the *Western Medical and Physical Journal*, of which this Journal is a continuation. Dr. Drake had anticipated the enterprise at Lexington by a year, and appropriately styled his publication the "*Western* Journal," and adopted for its motto the only representative of medicine as it was in the backwoods, "*E sylvis nuncius.*" But things have greatly changed with us in these five-and-twenty years. Medical journals have started up in most of the States around us, many of them to struggle on for a little season and then expire, but some of them give promise of becoming permanent. With this great increase of our serials it must be admitted that the necessity which formerly existed for an organ of the profession in these States has ceased to be felt in its original force, but still, we think, all will agree with us, that it is desirable that this by far the oldest journal in the West should be continued. Dating from its origin in Cincinnati, in 1827, it is now in its thirtieth year. It has survived its enterprising, sanguine projector, and is outliving a generation of men.

As we look over the long rows of volumes ranged on our shelves which have issued from the press under our supervision it is with any other feeling than one of pride that we contemplate our labors, and yet it is not without some gratifying assurance that these works have contributed, somewhat, to the diffusion and increase of medical knowledge. Our steady aim has always been, in the direction of the journals of which we have had charge, to advance and elevate our profession, and, by every means in our power, to promote its true interests. We have endeavored to make them representatives of a liberal, enlightened profession, and to avoid everything selfish, narrow, and sectional, remembering that the principles of truth and justice will remain after all our personal bickerings, and heartburnings, and petty interests have passed away and been forgotten. It has been our wish to exclude from our pages everything that is low, and to preserve a purity and dignity of style and manner becoming a learned profession. In the independent discharge of our duties we have sometimes been called upon to rebuke pretension and condemn error, and as a natural consequence have occasionally become involved in unpleasant controversies; but we have sought to conduct our disputes with temperance, and believe our pages have been kept free from personalities. Our relations with our brethren of the press have at all times been harmonious and we part with them all on good terms.

Of those who took a warm interest in the first Western journals of medicine and were the most frequent contributors to them when we became an editor, not one is now engaged in active professional life. Dr. WILLIAM H. RICHARDSON, who wrote for some of the earlier numbers of the Transylvania Journal, died in 1845. The Rev. Dr. JAMES BLYTHE, also an early contributor to that journal, had paid the debt of nature several years before. Dr. CALDWELL, the most voluminous of all its contributors, and Dr. COOKE, who was one of its first editors, have followed their colleagues "from sun-shine to the sunless land." Our earliest and most gifted associate in the editorship of this journal, the versatile, ready, active-minded, original Dr. DRAKE, rests, too, from his labors. Two only remain—the great Surgeon of Lexington, and the *Emeritus* Professor of Materia Medica and Botany in the University of Louisville—passing the evening of their lives in dignified retirement:

> Serus in coelum redeant, diuque
> Laetus intersint populo!

It is with no ordinary feelings of satisfaction that, after these long years of service, we resign our task into the guardianship of editors so able as those by whom it is now to be prosecuted. We need not commend them to our brethren; they are known to all, and by all it will be acknowledged, that they bring to the work every qualification requisite to eminent success. The Journal, under the name, now more appropriate, of the "*Louisville Medical and Surgical Review*," cannot fail to reach a degree of distinction greater than it has yet attained. We not only tender to our successors our best wishes, but pledge them our hearty co-operation. The following is their circular:—

*To the Medical Profession:—*

The undersigned take this mode of announcing that on the 1st day of May, they will commence editing and publishing a bi-monthly periodical entitled the "*Louisville Medical and Surgical Review*," to be devoted, 1st, To critical and analytical reviews of select medical and surgical works, both old and new; 2d, To original communications, such as essays on special diseases, medical and surgical statistics, brief histories of cases worthy of record, etc.; 3d, To extracts from American and Foreign Journals; and 4th, To the mention of such miscellaneous items of general interest as naturally collect upon an editor's table. In a word, they will endeavor to furnish a journal which shall contain what is practically useful to the largest number of professional men, giving matters of mere curiosity or opinion but slight consideration; and shall be strictly independent of any school, clique, or section. For this purpose they have engaged the services of a large number of regular contributors, in different parts of the Union, the mention of whose names will give assurance of an amount of allied editorial talent unsurpassed by any similar publication in the country. They have also established an agency in one of the European cities for the prompt procurement of all the more valuable English, French, and German publications, in order to present their readers with any new facts or suggestions which they may contain, without waiting for the slow process of selection, translation, and republication by Eastern journals.

The editors have undertaken this enteprise as a labor of love—love for the labor and for the glorious profession in which they are actively engaged. They do not expect, nor do they wish to make money by it; but they look to the physicians of the West and South to sustain them against loss.

Each number of the journal will consist of *one hundred and twenty-eight pages;* and will be sent punctually on the first day of every alternate month, to all who pay their subscriptions in advance. The subscription price is three dollars per annum.

All communications, books, pamphlets, etc., addressed to the junior editor, will meet with prompt attention.

S. D. GROSS, M. D.,
*Professor of Surgery in the University of Louisville.*
T. G. RICHARDSON, M. D.,
*Demonstrator of Anatomy in the University of Louisville.*

LOUISVILLE, JAN., 1856.

